# Effect of Coconut Milk, Cow Milk, and Soybean Oil on the Surface Roughness of Milled (PICN, RNC) and 3D-Printed Hybrid Resin–Ceramic: An In Vitro Study

**DOI:** 10.3390/polym18060670

**Published:** 2026-03-10

**Authors:** Seelassaya Leelaponglit, Awiruth Klaisiri, Chayanit Angkananuwat, Nantawan Krajangta

**Affiliations:** Division of Restorative and Esthetic Dentistry, Faculty of Dentistry, Thammasat University, Pathum Thani 12120, Thailand; seelassa@tu.ac.th (S.L.); dentton@tu.ac.th (A.K.); chayanit@tu.ac.th (C.A.)

**Keywords:** dietary media, hybrid resin ceramic, surface roughness

## Abstract

This in vitro study assessed the impact of coconut milk, cow milk, and soybean oil on the surface roughness (Ra) of two milled (polymer-infiltrated ceramic network (PICN), Vita Enamic (EN) and resin nanoceramic (RNC), Cerasmart (CS)) and 3D-printed (VarseoSmile Crown plus (VS)) hybrid resin–ceramic materials. Standardized rectangular specimens were prepared and subjected to cyclic immersion in the test media at 37 °C for 30 days to simulate dietary exposure. Surface roughness was measured pre- and post-aging, and statistical analysis was performed using two-way ANOVA and paired *t*-tests (α = 0.05). All media significantly increased Ra across all materials (*p* < 0.001). While coconut milk and soybean oil caused comparable roughening (Ra up to 0.155 µm), cow milk exhibited a material-specific impact. It roughened milled materials (EN and CS) (Ra: 0.147–0.154 µm) significantly more than the 3D-printed material (VS) (Ra: 0.126 µm) (*p* < 0.05). Notably, all post-aging Ra values remained below the clinical bacterial adhesion threshold of 0.2 µm. In conclusion, while all tested dietary media significantly degraded the surface topography of hybrid resin–ceramics, the 3D-printed hybrid resin–ceramic material demonstrated superior resistance to cow milk compared to milled alternatives. Nonetheless, plaque retention risks remain clinically acceptable for all tested materials.

## 1. Introduction

The rapid advancement of digital dentistry has been largely driven by continuous innovations in material science, particularly concerning the microstructural engineering and crystal growth mechanisms of ceramic components, which dictate the ultimate mechanical and physicochemical properties of dental restorations [1]. Meanwhile, a diverse array of hybrid resin–ceramic materials has emerged, designed to combine the high elastic modulus and aesthetics of traditional ceramics with the flexibility and ease of milling associated with cross-linked polymers. Among these, polymer-infiltrated ceramic networks (PICN), such as Vita Enamic (EN), and resin nanoceramics (RNC), such as Cerasmart (CS), have become staples in CAD-CAM restorative dentistry due to their excellent machinability and tooth-like wear characteristics. More recently, the emergence of 3D-printed hybrid resin–ceramic materials, such as VarseoSmile Crown plus (VS), has offered an additive and cost-effective manufacturing alternative. Although the mechanical properties of these materials are well documented, their long-term surface stability in various dietary environments remains unclear. Specifically, the complex interaction between their unique microstructures and lipid-based fluids requires further study.

The selection of these three materials for evaluation is based on their distinct architectural compositions. EN features a dual-network structure where a ceramic scaffold is infiltrated with a monomer. CS consists of a highly filled resin matrix with nanoceramic particles. In contrast, VS represents the latest generation of 3D-printing technology. It is characterized by an incremental layer-by-layer polymerization process that may result in cross-linking densities different from those of high-pressure milled blocks. Understanding how these structural variations influence surface degradation is vital for clinicians when selecting the appropriate material for long-term clinical success.

In the complex milieu of the oral cavity, restorative materials are subjected to continuous chemo-mechanical challenges. Surface integrity, quantified by roughness (Ra), is a critical determinant of clinical longevity; increased roughness not only compromises aesthetics but also accelerates biofilm accumulation, secondary caries, and antagonistic wear [2]. While the current literature provides a comprehensive understanding of hybrid material behavior under standard hydrolytic challenges, aqueous pH fluctuations, and thermal cycling [3,4]. A significant gap remains regarding their stability in organic dietary environments. Recent studies, such as those by Seelassaya et al. [3] have characterized the differential impacts of thermal and acidic aging on milled versus printed substrates, yet the potentially deleterious solvent and plasticizing effects of lipid-based emulsions have been largely overlooked. This finding provides significant novelty. The oral environment frequently encounters complex organic dietary components that act as penetrating solvents, potentially compromising the surfaces of resin-based restorations more severely than simple acid erosion [2,5,6]. Therefore, this study focuses on three culturally and dietarily relevant media: coconut milk (Chaokoh), cow milk (Bear Brand), and soybean oil (Angoon). Coconut milk, a staple in Southeast Asian diets, contains high concentration of medium chain triglycerides (MCTs) and lauric acid that can penetrate resin matrices. Cow milk is a complex animal-based emulsion containing proteins (casein/whey) and lactic acid, which may interact differently with the inorganic–organic material interfaces. Finally, soybean oil, a pure polyunsaturated fat, serves as a baseline for assessing the impact of concentrated lipids on the hydrolytic stability of the silane coupling agents and polymer chains [7].

As surface roughness (Ra) is directly linked to plaque accumulation, gingival irritation, and the aesthetic degradation of restorations, evaluating how these specific dietary components influence surface topography is essential [8]. Despite the growing adoption of 3D-printed hybrid resin–ceramic material, data comparing their chemical stability in organic solvents relative to milled counterparts remains limited. It is unclear whether the layer-by-layer structure of printed resins exhibits higher permeability to dietary lipids than the dense, interpenetrating networks of milled blocks. Therefore, this study aims to evaluate the surface roughness of milled materials and 3D-printed hybrid resin–ceramic materials following immersion in coconut milk, cow milk, and soybean oil. The null hypothesis is that neither the manufacturing method (milled vs. printed) nor the type of dietary medium will significantly alter the surface topography of the restorative materials.

## 2. Materials and Methods

### 2.1. Specimen Preparation

This in vitro study evaluated three commercial resin–ceramic hybrid materials representing subtractive (milled) and additive (3D-printed) manufacturing technologies. The materials included a PICN (VE; VITA Zahnfabrik, Bad Säckingen, Germany), RNC (CS; GC Dental Products, Tokyo, Japan), and a 3D-printed ceramic-filled hybrid resin (VS; BEGO, Bremen, Germany).

Sample size was calculated using statistical power analysis software (G*Power v. 3.1.9.2; Heinrich-Heine-Universität Düsseldorf, Düsseldorf, Germany). Based on data from a previous study on the surface degradation of dental composites in dietary solvents [9], an effect size of 0.30 was determined. To achieve a power of 80% and a significance level of 5%, a total sample size of 108 specimens (*n* = 36 per material group; *n* = 12 per subgroup) was required.

For the subtractive groups (EN and CS), standardized rectangular specimens (12 × 14 × 2 mm) were fabricated from their respective CAD/CAM blocks. Sectioning was performed using a precision low-speed diamond saw (Accutom-50; Struers, Ballerup, Denmark) under continuous water cooling to prevent thermal damage to the polymer matrix.

For the additive group (VS), specimens of identical dimensions (12 × 14 × 2 mm) were designed using CAD software (DentalCAD 3.2 Elefsina; exocad GmbH, Darmstadt, Germany) and exported in Standard Tessellation Language (STL) format. Fabrication was executed using a Digital Light Processing (DLP) 3D printer (Varseo XS; BEGO, Bremen, Germany) equipped with a 385 nm UV light-emitting diode (LED). Printing parameters were standardized to a layer thickness of 50 µm. Following printing, specimens underwent a standardized post-processing protocol to ensure optimal degree of conversion and biocompatibility:

1. Cleaning: A two-stage immersion in 96% ethanol using an ultrasonic bath, consisting of a 3 min pre-wash followed by a 2 min main wash to eliminate unpolymerized surface resin.
2. Drying: Specimens were dried using compressed air.
3. Post-Curing: Final polymerization was achieved using a high-performance flash-curing unit (Otoflash G171; NK-Optik, Baierbrunn, Germany) with 2 × 1500 flashes under a nitrogen atmosphere to prevent oxygen inhibition layer formation.

To simulate clinical delivery and standardize baseline surface topography, all specimens underwent a uniform finishing protocol using an automated microprocessor-controlled grinding and polishing machine (LaboPol-30; Struers, Ballerup, Denmark). The sequence utilized silicon carbide (SiC) abrasive papers of P1200 and P4000 grit (Water Proof SiC Paper; Struers, Ballerup, Denmark) under continuous water irrigation. The machine operated at a pressure of 5 N, with a polishing disc speed of 300 rpm and a specimen holder speed of 150 rpm for 20 s.

### 2.2. Artificial Aging with Dietary Challenge

A randomization protocol was employed to assign specimens from each material category to their respective study subgroups. Prior to artificial aging, baseline surface roughness values were statistically analyzed to ensure homogeneity across all groups (*p* > 0.05), confirming that no initial surface differences existed between the test groups.

A total of 108 specimens were randomly allocated into three subgroups based on the dietary immersion medium:

1. Coconut milk (Choakoh; TCC-Choakoh, Bangkok, Thailand): This is characterized by high saturated fat content, specifically medium-chain triglycerides (MCT) such as lauric acid.
2. Cow milk (Bear Brand UHT; Nestlé (Thai) Ltd., Bangkok, Thailand): An emulsion containing lactose and proteins.
3. Soybean oil (Angoon; Thai Vegetable Oil PCL, Bangkok, Thailand): This is composed of 100% refined polyunsaturated fats.

To simulate intermittent clinical dietary exposure, a cyclic immersion protocol was adopted, modifying methods previously described by Seelassaya et al. [3]. Specimens were immersed in fresh preparations of the respective food media for 15 min, three times daily (morning, afternoon, and evening) for a duration of 30 days. This specific frequency (15 min, 3 times daily) was selected to simulate the transient contact of food during meals, a paradigm supported by established dietary aging models [10,11]. The accelerated media laboratory aging 30-day regimen yielded a cumulative active exposure time of approximately 1350 min (22.5 h). Considering that intraoral exposure to dietary lipids is transient and moderated by salivary clearance mechanisms [9,12,13]. With estimated contact durations of approximately 15–30 min per day during typical meal consumption, the present protocol may theoretically correspond to approximately 45–90 days (about 1.5–3 months) of cumulative dietary exposure. All immersions were conducted at a physiologic temperature of 37 °C in a laboratory incubator. Between dietary cycles, specimens were stored in artificial saliva to simulate the oral resting state and facilitate potential buffering effects. The food media were replaced daily to prevent microbial fermentation or rancidity.

The artificial saliva, the immersion medium at resting state, was prepared according to a previously established protocol [14]. The chemical composition was formulated to mimic the viscosity and electrolyte balance of natural saliva. All reagents were of analytical grade (Sigma-Aldrich, St. Louis, MO, USA) and included: 10 g/L sodium carboxymethyl cellulose (Na-CMC), 2 g/L methyl-p-hydroxybenzoate, 0.804 g/L dipotassium hydrogen phosphate (K_2_HPO_4_), 0.625 g/L potassium chloride (KCl), 0.326 g/L potassium dihydrogen phosphate (KH_2_PO_4_), 0.166 g/L calcium chloride dihydrate (CaCl_2_·2H_2_O), and 0.059 g/L magnesium chloride hexahydrate (MgCl_2_·6H_2_O). To prepare the artificial saliva, the components were sequentially dissolved in 1000 mL of deionized water. The mixture was homogenized using a magnetic stirrer at room temperature for six hours to ensure complete dissolution. The final solution exhibited a physiological pH of 6.8–7.0, actively sustained by the phosphate buffer system (K_2_HPO_4_/KH_2_PO_4_) to accurately simulate human resting saliva. During the artificial aging period, the saliva substitute was maintained at a physiologic temperature of 37 °C. To prevent microbial proliferation and maintain chemical stability, the solution was freshly prepared and replaced every 24 h.

### 2.3. Surface Roughness (Ra) Measurement

To minimize observer bias, the operator performing the surface roughness measurements was blinded to both the material type and the aging condition throughout the data acquisition process.

Quantitative surface analysis was performed using a non-contact optical Micro-Coordinate Measurement Machine (Alicona InfiniteFocus; Alicona Imaging GmbH, Graz, Austria). Measurements were taken at 100× magnification with direct illumination. Surface roughness parameter, arithmetical mean height (Ra), was calculated in accordance with ISO 21920-2:2021 [15]. Three random areas were scanned per specimen, and the mean value was recorded as the Ra.

### 2.4. Scanning Electron Microscopy (SEM)

To qualitatively characterize the surface alteration, one representative specimen from each material–medium combination was selected for topographic analysis using Scanning Electron Microscopy (JCM-6000; JEOL Ltd., Akishima, Tokyo, Japan) at 2000 magnifications with an accelerating voltage of 10 kV. Before imaging, specimens were mounted on aluminum stubs and sputter-coated with gold to enhance conductivity and prevent charging artifacts.

### 2.5. Statistical Analysis

Data were analyzed using SPSS software (IBM SPSS Statistics, Version 26.0; IBM Corp., Armonk, NY, USA). Normality and homogeneity of variance were verified using the Kolmogorov–Smirnov and Levene’s tests, respectively. A two-way analysis of variance (ANOVA) was employed to evaluate the interaction between “Material type” and “Dietary medium”. Significant interactions were further analyzed using Tukey’s post hoc test. Paired *t*-tests were used to compare pre- and post-aging roughness values within each group. All tests were conducted at a significance level of α = 0.05 and a 95% confidence interval.

## 3. Results

The result of surface roughness (Ra) revealed that dietary aging had a significant degradative effect on the surface topography of all tested hybrid resin–ceramic materials. As presented in Table 1, a statistically significant increase in Ra was observed for every material group following the 30-day cyclic immersion protocol, regardless of the dietary medium used (*p* < 0.001).

**Figure 1:**
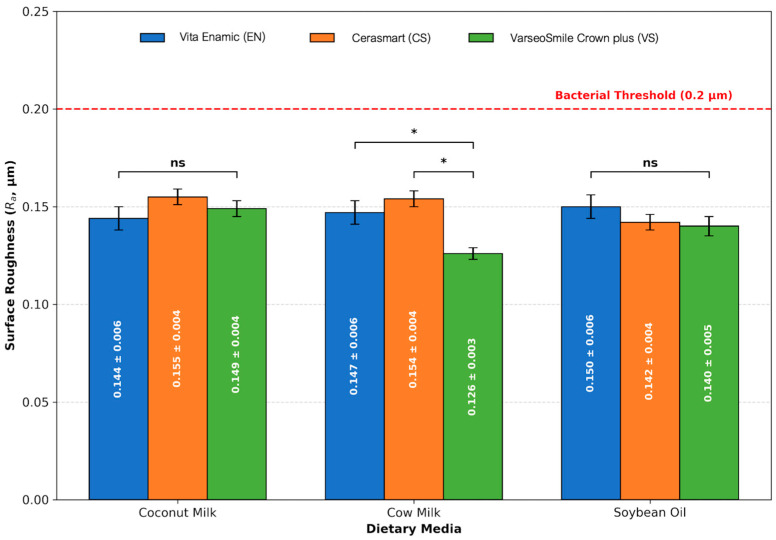
Comparison of mean post-aging surface roughness (Ra) of hybrid resin–ceramic materials in various dietary media. The red dashed line indicates the clinical bacterial adhesion threshold (0.2 μm). Horizontal brackets represent pairwise comparisons between materials within each specific dietary medium. The abbreviation “ns” denotes no statistically significant difference (*p* > 0.05), while the asterisk (*) indicates a statistically significant difference (*p* < 0.05). All tested groups remained below the critical threshold for plaque accumulation. Error bars signify standard deviations.

**Figure 2:**
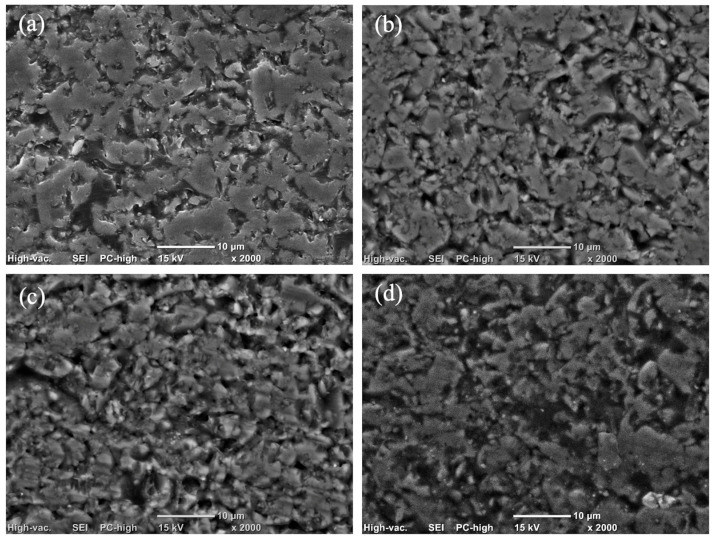
Scanning electron microscopy (SEM) micrographs showing the surface topography of EN under different experimental conditions: (**a**) before aging; (**b**) after immersion in coconut milk; (**c**) after immersion in cow milk; and (**d**) after immersion in soybean oil.

**Figure 3:**
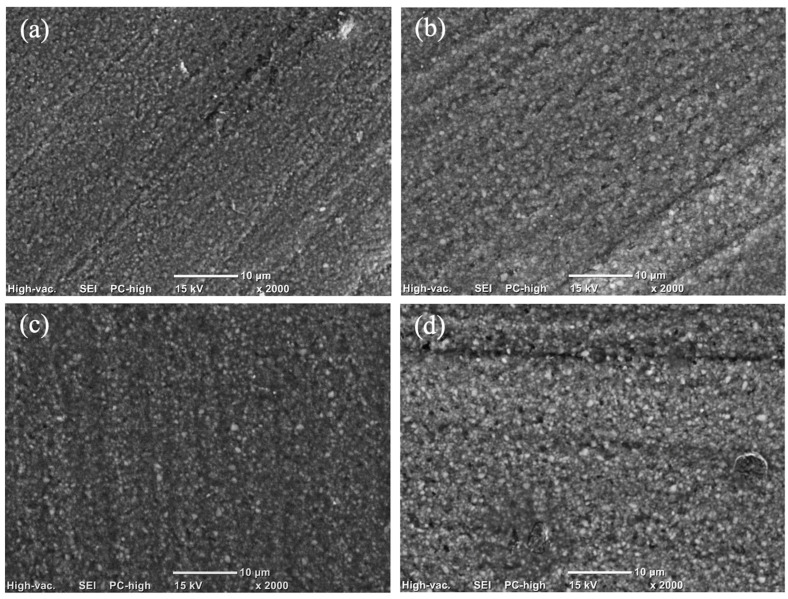
Scanning electron microscopy (SEM) micrographs showing the surface topography of CS under different experimental conditions: (**a**) before aging; (**b**) after immersion in coconut milk; (**c**) after immersion in cow milk; and (**d**) after immersion in soybean oil.

**Figure 4:**
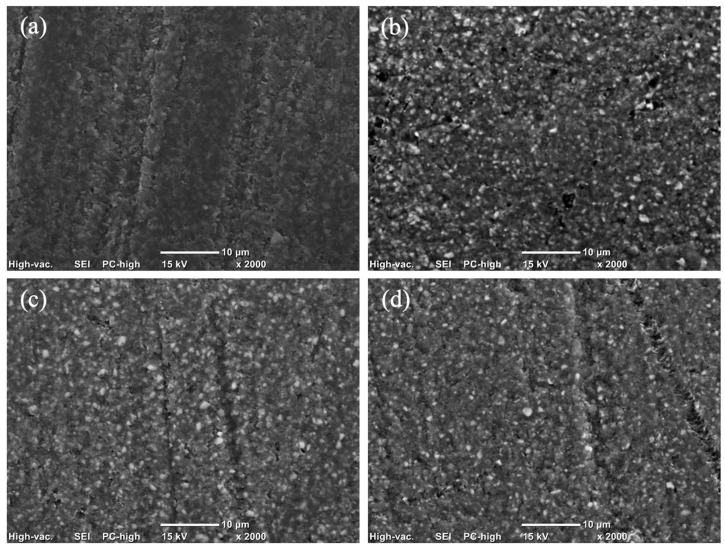
Scanning electron microscopy (SEM) micrographs showing the surface topography of VS under different experimental conditions: (**a**) before aging; (**b**) after immersion in coconut milk; (**c**) after immersion in cow milk; and (**d**) after immersion in soybean oil.

The magnitude of this increase varied, with post-aging values generally shifting from a baseline range of 0.095–0.120 µm to a roughened state of 0.126–0.155 µm. However, it is noteworthy that despite these significant alterations, the mean Ra values for all groups remained below the established threshold for bacterial retention (Ra < 0.2 µm) as Figure 1. Scanning electron microscope (SEM) images are presented in Figure 2, Figure 3 and Figure 4.

## 4. Discussion

The present study rejects the null hypothesis, as all tested dietary media induced statistically significant surface roughening (*p* < 0.001) across both milled and 3D-printed hybrid resin–ceramic materials. Qualitative SEM observations were consistent with these findings, revealing surface irregularities and micro-topographical changes that aligned with the observed Ra trends.

Although all materials exhibited increased surface roughness following the 30-day immersion protocol, the 3D-printed hybrid resin (VS) demonstrated significantly lower post-aging Ra values (0.126 ± 0.003 µm) compared to the RNC (CS: 0.154 ± 0.004 µm) and the polymer-infiltrated ceramic network (EN: 0.147 ± 0.006 µm). These results align with recent findings by Seelassaya et al. (2025), who reported that 3D-printed hybrid resin–ceramic (VS) exhibited superior stability compared to milled counterparts under thermal and pH cycling [3]. However, their study attributed the degradation of milled materials primarily to thermal expansion mismatch at the ceramic–polymer interface, whereas the present investigation implicates chemical interaction with dietary lipids as a distinct degradation mechanism. This suggests that while 3D-printed resins demonstrate thermal resilience, they may exhibit differential vulnerabilities to specific organic solvents encountered during dietary exposure.

The findings revealed a significant material-dependent response to dietary challenge, particularly when specimens were exposed to cow milk. The heightened susceptibility of milled materials (EN and CS) to cow milk-induced degradation is likely linked to the silane-coupling interface. In resin nanoceramics such as CS, a high volume of inorganic fillers is bonded to the resin matrix via silane coupling agents. Cow milk is a complex aqueous emulsion containing proteins (casein and whey), lactose and lactic acid. These components may facilitate hydrolytic degradation. Even in small concentrations, lactic acid, could potentially catalyze the hydrolysis of siloxane bonds (Si-O-Si) at the filler–matrix interface [16,17,18]. The gradual hydrolysis of these interfacial bonds likely facilitates filler dislodgement. This manifests as increased surface roughness, as evidenced in Figure 2c and Figure 3c. Similarly, the dual-network structure of EN relies on the integrity of the interface between the ceramic scaffold and the infiltrating polymer. The aqueous nature of milk might preferentially penetrate this interface, compromising interfacial adhesion and accelerating surface erosion [19].

Conversely, the 3D-printed hybrid resin–ceramic (VS) exhibited relative stability in cow milk, showing the lowest post-aging roughness (0.126 ± 0.003 µm) among all groups. This stability may be explained by the high concentration of casein in cow milk. Casein is an amphiphilic phosphoprotein capable of rapid adsorption onto dental materials [20]. It is hypothesized that casein adsorbs onto solid substrates via electrostatic and hydrophobic interactions, forming a dense proteinaceous conditioning film. This film may function as a sacrificial barrier, attenuating direct solvent–polymer interactions [21,22,23]. Furthermore, the fat globules in milk emulsions may provide a boundary lubrication effect, reducing frictional shear stress on the material surface during the immersion cycles [21,24]. For 3D-printed materials, which typically exhibit higher surface free energy due to residual unpolymerized monomers, this protein adsorption may be thermodynamically favorable [25,26]. This effectively seals surface micro-porosities and mitigates the diffusion-driven plasticization seen with lipids [24].

A critical finding was the pronounced susceptibility of the 3D-printed material (VS) to coconut milk, reaching its highest post-aging roughness (0.149 ± 0.004 µm). This behavior could be attributed to the molecular kinetics of lauric Acid (C12:0), the predominant saturated fatty acid in coconut milk. Unlike the bulky long-chain triglycerides (LCTs) found in soybean oil, Lauric acid is a medium-chain triglyceride (MCT) with a lower molecular weight and smaller hydrodynamic radius [27]. According to free volume theory, these smaller molecules possess a higher diffusion coefficient, allowing them to penetrate the polymer network of the 3D-printed resin more readily than larger lipid chains. Once absorbed, these MCTs likely act as external plasticizers, neutralizing secondary van der Waals forces between polymer chains and inducing localized swelling [28]. In a 3D-printed structure, this swelling is hypothesized to be anisotropic, occurring preferentially at the layer interfaces, which manifests as increased surface peaks and valleys (Ra).

Conversely, the PICN material (EN) displayed its highest roughness increase in soybean oil (0.095 to 0.150 µm) and exhibited a greater degree of inter-filler degradation, as illustrated in Figure 2d. Unlike the inert saturated fats in coconut milk, soybean oil is rich in polyunsaturated linoleic acid (C18:2). It is proposed that unsaturated fatty acids are highly susceptible to auto-oxidation, a process accelerated at body temperature that generates lipid hydroperoxides and reactive free radicals, such as alkoxyl and peroxyl [29]. These radicals can abstract hydrogen atoms from the polymer backbone and may initiate chain scission in the acrylate network (UDMA/TEGDMA) that serves as the structural matrix for the ceramic scaffold. The resultant polymer degradation manifests as filler plucking or micro-pitting at the ceramic–polymer interface, increasing the Ra surface observed in aged materials.

This investigation demonstrates that the interaction between restorative materials and dietary components is more complex than previously understood. Manufacturing methods, chemical compositions, and environmental factors all contribute to degradation patterns. While dietary media induced statistically significant increases in surface roughness, these changes may not be clinically meaningful in the short term regarding bacterial colonization. From a biocompatibility standpoint, all evaluated materials maintained surface roughness values below the established threshold for pathogenic bacterial adhesion (Ra < 0.2 µm). This confirms their short-term clinical viability despite their measurable chemical susceptibility [8]. Regarding diet-specific selection, patients who consume diets high in medium-chain triglycerides (MCTs), such as those on ketogenic diets or who frequently consume coconut-based cuisines, may experience accelerated surface aging and degradation of 3D-printed restorations.

The present study assessed chemical aging in isolation and did not include mechanical loading or abrasion. The proposed degradation pathways are inferred from surface roughness and SEM observations alone. In the absence of direct chemical or interfacial analyses, these findings should be regarded as plausible hypotheses rather than confirmed mechanisms. Future investigations integrating combined chemo-mechanical aging protocols and advanced surface analytical techniques, such as FTIR, XPS, sorption–solubility analysis) are warranted to validate the proposed mechanisms and strengthen clinical extrapolation.

## 5. Conclusions

Within the limitations of this in vitro study. The following conclusions were drawn:

Main findings:

(1) All dietary media, including coconut milk, cow milk, and soybean oil, induced a statistically significant increase in surface roughness across all hybrid resin–ceramic materials. However, post-aging values remained safely below the clinical threshold for bacterial adhesion (Ra < 0.2 µm).
(2) Material roughening was medium-dependent. The 3D-printed resin–ceramic was most vulnerable to coconut milk, whereas the milled resin–ceramics were significantly more susceptible to cow milk.

Clinical recommendations:

Material selection should align with the patient dietary habits. 3D-printed restorations are less optimal for patients with high coconut milk intake, while milled resin–ceramics are more susceptible to dairy. Although short-term media-induced roughening is clinically acceptable, the resulting chemical softening may accelerate in vivo wear under masticatory forces. Therefore, routine clinical recalls, re-evaluation, and periodic repolishing are advised to ensure long-term restoration integrity.

## Figures and Tables

**Table 1:** Mean surface roughness (Ra) and standard deviation of hybrid resin–ceramic materials before and after artificial aging in various dietary media.

Material	Dietary Media	Surface Roughness, Ra (µm)		*p*-Value of Paired *t*-Test
		Before Aging	After Aging	
**EN** **(milled)**	**Coconut milk**	0.105 ± 0.002 ^ABC,a^	0.144 ± 0.006 ^AB,b^	<0.001
	**Cow milk**	0.096 ± 0.004 ^B,a^	0.147 ± 0.006 ^A,b^	<0.001
	**Soybean oil**	0.095 ± 0.004 ^B,a^	0.150 ± 0.006 ^A,b^	<0.001
**CS** **(milled)**	**Coconut milk**	0.118 ± 0.002 ^CD,a^	0.155 ± 0.004 ^A,b^	<0.001
	**Cow milk**	0.120 ± 0.003 ^D,a^	0.154 ± 0.004 ^A,b^	<0.001
	**Soybean oil**	0.113 ± 0.003 ^ACD,a^	0.142 ± 0.004 ^AB,b^	<0.001
**VS** **(3D-printed)**	**Coconut milk**	0.110 ± 0.004 ^ABCD,a^	0.149 ± 0.004 ^A,b^	<0.001
	**Cow milk**	0.102 ± 0.004 ^A,a^	0.126 ± 0.003 ^B,b^	<0.001
	**Soybean oil**	0.114 ± 0.005 ^ABCD,a^	0.140 ± 0.005 ^AB,b^	<0.001

Different uppercase letters (A to D) within a column indicate significant differences among the material and dietary media groups (Tukey’s HSD). Different lowercase letters (a and b) indicate significant differences between the “Before” and “After” conditions (paired *t*-tests).

## Data Availability

Data are contained within the article.
